# Tetra­kis(2-amino­thia­zole-κ*N* ^3^)dichloridocadmium(II)

**DOI:** 10.1107/S1600536809051770

**Published:** 2009-12-04

**Authors:** Chong-Hyeak Kim, Inn Hoe Kim

**Affiliations:** a Center for Chemical Analysis, Korea Research Institute of Chemical Technology, PO Box 107, Yuseong, Daejeon 305-600, Republic of Korea; b Department of Chemistry, Konyang University, Nonsan 320-711, Republic of Korea

## Abstract

In the title complex, [CdCl_2_(C_3_H_4_N_2_S)_4_],the Cd^II^ atom has an *trans*-Cl_2_N_4_ octa­hedral coordination geometry defined by four N atoms derived from the four 2-amino­thia­zole ligands and two Cl atoms. The amino groups participate in intra- and inter­molecular N—H⋯N and N—H⋯Cl hydrogen bonding that stabilizes both the mol­ecular and crystal structures.

## Related literature

For the coordination properties of heterocycles, see: Raper (1994 ▶); Karlin & Zubieta (1983 ▶). For the structures of related amino­thia­zole complexes, see: Batı *et al.* (2006 ▶); Davarski *et al.* (1996 ▶); Macíček & Davarski (1993 ▶); Maniukiewicz (2004 ▶); Raper *et al.* (1981 ▶); Suh *et al.* (2005 ▶, 2007 ▶, 2009 ▶).
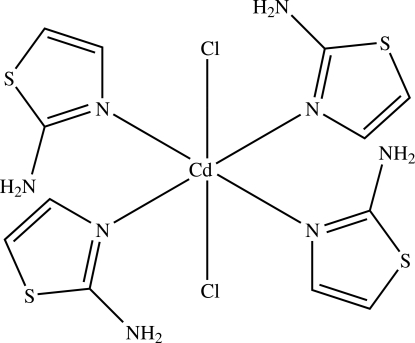


## Experimental

### 

#### Crystal data

[CdCl_2_(C_3_H_4_N_2_S)_4_]
*M* *_r_* = 583.87
Monoclinic, 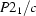

*a* = 8.6056 (1) Å
*b* = 15.2838 (2) Å
*c* = 16.2097 (2) Å
β = 103.605 (1)°
*V* = 2072.18 (4) Å^3^
*Z* = 4
Mo *K*α radiation
μ = 1.73 mm^−1^
*T* = 296 K
0.40 × 0.19 × 0.08 mm

#### Data collection

Bruker SMART CCD area-detector diffractometer
Absorption correction: multi-scan (*SADABS*; Bruker, 2001 ▶) *T*_min_ = 0.544, *T*_max_ = 0.870
21163 measured reflections
5159 independent reflections
4532 reflections with *I* > 2σ(*I*)
*R*_int_ = 0.020

#### Refinement

*R*[*F* ^2^ > 2σ(*F* ^2^)] = 0.021
*wR*(*F* ^2^) = 0.052
*S* = 1.05
5159 reflections
244 parameters
H-atom parameters constrained
Δρ_max_ = 0.36 e Å^−3^
Δρ_min_ = −0.30 e Å^−3^

### 

Data collection: *SMART* (Bruker, 2001 ▶); cell refinement: *SAINT* (Bruker, 2001 ▶); data reduction: *SAINT*; program(s) used to solve structure: *SHELXS97* (Sheldrick, 2008 ▶); program(s) used to refine structure: *SHELXL97* (Sheldrick, 2008 ▶); molecular graphics: *SHELXTL* (Sheldrick, 2008 ▶); software used to prepare material for publication: *SHELXTL*.

## Supplementary Material

Crystal structure: contains datablocks global, I. DOI: 10.1107/S1600536809051770/tk2590sup1.cif

Structure factors: contains datablocks I. DOI: 10.1107/S1600536809051770/tk2590Isup2.hkl

Additional supplementary materials:  crystallographic information; 3D view; checkCIF report

## Figures and Tables

**Table 1:** Hydrogen-bond geometry (Å, °)

*D*—H⋯*A*	*D*—H	H⋯*A*	*D*⋯*A*	*D*—H⋯*A*
N16—H16*A*⋯N23	0.86	2.63	3.277 (2)	133
N16—H16*A*⋯Cl1	0.86	2.81	3.3903 (19)	126
N16—H16*B*⋯Cl2^i^	0.86	2.52	3.2941 (18)	151
N26—H26*A*⋯Cl2	0.86	2.41	3.1722 (17)	149
N26—H26*B*⋯Cl1^ii^	0.86	2.51	3.3300 (16)	161
N36—H36*A*⋯N43	0.86	2.61	3.324 (2)	142
N36—H36*B*⋯Cl1^iii^	0.86	2.63	3.3810 (18)	147
N46—H46*A*⋯Cl1	0.86	2.44	3.2135 (18)	150
N46—H46*B*⋯N36^iv^	0.86	2.56	3.417 (2)	177

Symmetry codes: (i) 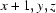; (ii) 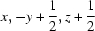; (iii) 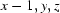; (iv) 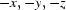.

**Fig. 1.**
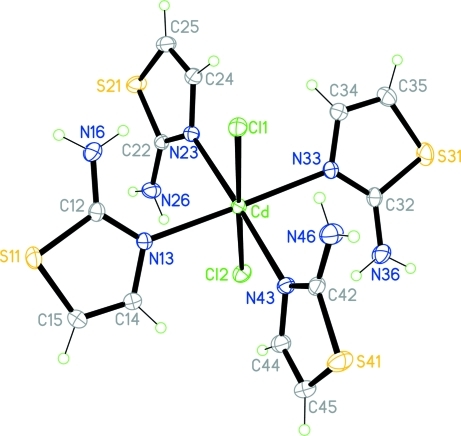
The molecular structure of (I), showing 30% probability displacement ellipsoids and the atom-numbering scheme.

**Fig. 2.**
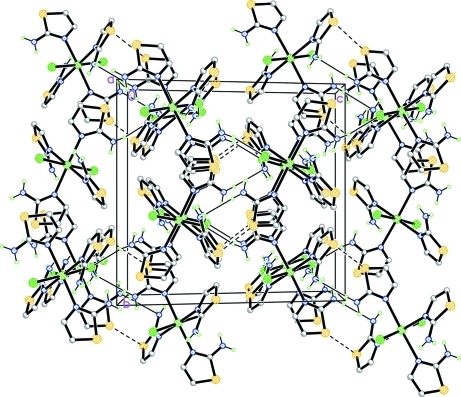
A view of the unit cell contents of (I). The C–H atoms have been omitted for reasons of clarity (dashed lines).

## supplementary crystallographic information

### Comment

Some heterocyclic organic compounds have biologically useful properties, having anti-tumour, anti-fungal, and anti-infection activities. Amongst these, aminothiazoles are an important type of N,S-containing heterocycle (Raper, 1994). The N and S atoms play a key role in the coordination of metals at the active sites of various metallobiomelecules (Karlin & Zubieta, 1983). The crystal structures of aminothiazole complexes have attracted recent interest (Suh *et al.*, 2005, 2007, 2009; Batı *et al.*, 2006; Davarski *et al.*, 1996; Macíček & Davarski, 1993; Maniukiewicz, 2004; Raper *et al.*, 1981). Herein, we report the synthesis and crystal structure of the title complex, (I).

As shown in Fig. 1, the complex (I) comprises discrete Cd(C_3_H_4_N_2_S)_4_Cl_2_ molecules. The octahedral Cd^II^ coordination environment is defined by four N atoms derived from four neutral monodentate 2-aminothiazole ligands and two Cl atoms [Cd—Cl = 2.6294 (5) and 2.6560 (4) Å, and Cd—N = 2.3569 (14)-2.4432 (14) Å]. The Cl atoms occupy *trans* positions. The amino groups participate in intra- and inter-molecular N—H···N and N—H···Cl hydrogen bonds (Table 1). In the crystal structure molecules are interconnected by these interactions into a three-dimensional hydrogen bond network (Fig. 2).

### Experimental

A water–ethanol (1:1) solution (40 ml) of 2-aminothiazole (5 mmol) was added dropwise to a water–ethanol (1:1) solution (40 ml) of CdCl_2_.2.5H_2_O (2 mmol) with stirring. The small amount of precipitates formed from the mixed solution were filtered off. The filtered solution was allowed to stand at room temperature. After several days, yellow blocks were obtained. Analysis found: C 24.95, H 2.74, N 19.11, S 21.72, Cd 19.30%; C_12_H_16_CdCl_2_N_8_S_4_ requires: C 24.68, H 2.76, N 19.20, S 21.96, Cl 12.14, Cd 19.25%.

### Refinement

Positional parameters for the H atoms were calculated geometrically and constrained to ride on their attached atoms with C—H = 0.93 Å and N—H = 0.86 Å, and with *U*_iso_(H) = 1.2*U*_eq_(C, N).

### Crystal data

**Table:**

[CdCl_2_(C_3_H_4_N_2_S)_4_]	*F*(000) = 1160
*M**_r_* = 583.87	*D*_x_ = 1.872 Mg m^−^^3^*D*_m_ = 1.87 Mg m^−^^3^*D*_m_ measured by flotation method
Monoclinic, *P*2_1_/*c*	Mo *K*α radiation, λ = 0.71073 Å
Hall symbol: -P 2ybc	Cell parameters from 5290 reflections
*a* = 8.6056 (1) Å	θ = 2.7–28.3°
*b* = 15.2838 (2) Å	µ = 1.73 mm^−^^1^
*c* = 16.2097 (2) Å	*T* = 296 K
β = 103.605 (1)°	Block, yellow
*V* = 2072.18 (4) Å^3^	0.40 × 0.19 × 0.08 mm
*Z* = 4	

### Data collection

**Table:**

Bruker SMART CCD area-detector diffractometer	5159 independent reflections
Radiation source: fine-focus sealed tube	4532 reflections with *I* > 2σ(*I*)
graphite	*R*_int_ = 0.020
φ and ω scans	θ_max_ = 28.4°, θ_min_ = 1.9°
Absorption correction: multi-scan (*SADABS*; Bruker, 2001)	*h* = −11→11
*T*_min_ = 0.544, *T*_max_ = 0.87	*k* = −20→20
21163 measured reflections	*l* = −20→21

### Refinement

**Table:**

Refinement on *F*^2^	Primary atom site location: structure-invariant direct methods
Least-squares matrix: full	Secondary atom site location: difference Fourier map
*R*[*F*^2^ > 2σ(*F*^2^)] = 0.021	Hydrogen site location: inferred from neighbouring sites
*wR*(*F*^2^) = 0.052	H-atom parameters constrained
*S* = 1.05	*w* = 1/[σ^2^(*F*_o_^2^) + (0.0234*P*)^2^ + 0.6132*P*] where *P* = (*F*_o_^2^ + 2*F*_c_^2^)/3
5159 reflections	(Δ/σ)_max_ < 0.001
244 parameters	Δρ_max_ = 0.36 e Å^−^^3^
0 restraints	Δρ_min_ = −0.30 e Å^−^^3^

### Special details

**Table:**

Geometry. All e.s.d.'s (except the e.s.d. in the dihedral angle between two l.s. planes) are estimated using the full covariance matrix. The cell e.s.d.'s are taken into account individually in the estimation of e.s.d.'s in distances, angles and torsion angles; correlations between e.s.d.'s in cell parameters are only used when they are defined by crystal symmetry. An approximate (isotropic) treatment of cell e.s.d.'s is used for estimating e.s.d.'s involving l.s. planes.
Refinement. Refinement of *F*^2^ against ALL reflections. The weighted *R*-factor *wR* and goodness of fit *S* are based on *F*^2^, conventional *R*-factors *R* are based on *F*, with *F* set to zero for negative *F*^2^. The threshold expression of *F*^2^ > σ(*F*^2^) is used only for calculating *R*-factors(gt) *etc*. and is not relevant to the choice of reflections for refinement. *R*-factors based on *F*^2^ are statistically about twice as large as those based on *F*, and *R*- factors based on ALL data will be even larger.

### Fractional atomic coordinates and isotropic or equivalent isotropic displacement parameters (Å^2^)

**Table:**

	*x*	*y*	*z*	*U*_iso_*/*U*_eq_	
Cd	0.237289 (14)	0.127887 (8)	0.242806 (7)	0.02555 (5)	
Cl1	0.41984 (5)	0.15665 (3)	0.13431 (3)	0.03443 (10)	
Cl2	0.05073 (6)	0.10433 (3)	0.34816 (3)	0.03723 (11)	
S11	0.72017 (6)	−0.00335 (3)	0.42080 (3)	0.04305 (13)	
C12	0.6028 (2)	0.07360 (12)	0.35499 (11)	0.0312 (4)	
N13	0.44963 (17)	0.05509 (9)	0.33585 (9)	0.0304 (3)	
C14	0.4223 (2)	−0.02238 (12)	0.37465 (12)	0.0369 (4)	
H14A	0.3200	−0.0453	0.3682	0.044*	
C15	0.5505 (3)	−0.06232 (13)	0.42170 (13)	0.0432 (5)	
H15	0.5485	−0.1146	0.4508	0.052*	
N16	0.6685 (2)	0.14549 (12)	0.33020 (12)	0.0482 (5)	
H16A	0.6089	0.1839	0.2992	0.058*	
H16B	0.7702	0.1532	0.3453	0.058*	
S21	0.44426 (7)	0.38898 (3)	0.41822 (3)	0.04409 (13)	
C22	0.3558 (2)	0.28774 (11)	0.39147 (11)	0.0292 (4)	
N23	0.34496 (18)	0.26409 (9)	0.31227 (9)	0.0295 (3)	
C24	0.4114 (2)	0.32847 (12)	0.27081 (12)	0.0367 (4)	
H24A	0.4153	0.3226	0.2142	0.044*	
C25	0.4692 (3)	0.39916 (14)	0.31605 (13)	0.0440 (5)	
H25	0.5160	0.4468	0.2956	0.053*	
N26	0.3052 (2)	0.23949 (10)	0.44936 (10)	0.0400 (4)	
H26A	0.2620	0.1892	0.4356	0.048*	
H26B	0.3159	0.2589	0.5002	0.048*	
S31	−0.24063 (6)	0.26745 (4)	0.06541 (4)	0.04824 (14)	
C32	−0.1262 (2)	0.18601 (12)	0.12468 (11)	0.0327 (4)	
N33	0.02729 (18)	0.20338 (10)	0.14653 (9)	0.0311 (3)	
C34	0.0573 (2)	0.28381 (12)	0.11430 (12)	0.0384 (4)	
H34A	0.1601	0.3066	0.1230	0.046*	
C35	−0.0703 (3)	0.32678 (14)	0.06998 (13)	0.0467 (5)	
H35	−0.0670	0.3814	0.0451	0.056*	
N36	−0.1933 (2)	0.10964 (11)	0.14150 (12)	0.0458 (4)	
H36A	−0.1340	0.0685	0.1683	0.055*	
H36B	−0.2950	0.1024	0.1254	0.055*	
S41	0.07311 (8)	−0.15017 (3)	0.08737 (4)	0.05010 (14)	
C42	0.1437 (2)	−0.04377 (12)	0.10337 (11)	0.0319 (4)	
N43	0.13773 (18)	−0.01062 (9)	0.17752 (9)	0.0309 (3)	
C44	0.0725 (2)	−0.07152 (12)	0.22319 (12)	0.0385 (4)	
H44A	0.0581	−0.0591	0.2771	0.046*	
C45	0.0316 (3)	−0.14861 (13)	0.18614 (13)	0.0446 (5)	
H45	−0.0129	−0.1948	0.2101	0.054*	
N46	0.1983 (2)	−0.00158 (11)	0.04363 (10)	0.0455 (4)	
H46A	0.2329	0.0512	0.0524	0.055*	
H46B	0.1987	−0.0272	−0.0035	0.055*	

### Atomic displacement parameters (Å^2^)

**Table:**

	*U*^11^	*U*^22^	*U*^33^	*U*^12^	*U*^13^	*U*^23^
Cd	0.02192 (7)	0.02708 (7)	0.02791 (7)	0.00039 (5)	0.00638 (5)	0.00172 (5)
Cl1	0.0292 (2)	0.0456 (3)	0.0302 (2)	−0.00291 (19)	0.01048 (17)	0.00135 (18)
Cl2	0.0292 (2)	0.0449 (3)	0.0411 (2)	−0.00374 (19)	0.01530 (19)	−0.0022 (2)
S11	0.0319 (3)	0.0432 (3)	0.0476 (3)	0.0073 (2)	−0.0034 (2)	0.0046 (2)
C12	0.0268 (9)	0.0340 (9)	0.0320 (9)	0.0029 (7)	0.0052 (7)	−0.0023 (7)
N13	0.0257 (7)	0.0313 (8)	0.0331 (8)	0.0016 (6)	0.0047 (6)	0.0035 (6)
C14	0.0342 (10)	0.0359 (10)	0.0395 (10)	−0.0050 (8)	0.0063 (8)	0.0053 (8)
C15	0.0451 (12)	0.0348 (10)	0.0462 (11)	0.0008 (9)	0.0033 (9)	0.0097 (9)
N16	0.0252 (9)	0.0486 (10)	0.0682 (12)	−0.0030 (7)	0.0054 (8)	0.0154 (9)
S21	0.0559 (3)	0.0367 (3)	0.0391 (3)	−0.0151 (2)	0.0100 (2)	−0.0074 (2)
C22	0.0275 (9)	0.0268 (8)	0.0313 (9)	0.0013 (7)	0.0028 (7)	−0.0005 (7)
N23	0.0310 (8)	0.0275 (7)	0.0295 (7)	−0.0006 (6)	0.0063 (6)	0.0009 (6)
C24	0.0377 (10)	0.0407 (11)	0.0310 (9)	−0.0061 (8)	0.0066 (8)	0.0035 (8)
C25	0.0495 (13)	0.0413 (11)	0.0411 (11)	−0.0136 (9)	0.0103 (9)	0.0042 (9)
N26	0.0550 (11)	0.0371 (9)	0.0281 (8)	−0.0106 (8)	0.0101 (7)	−0.0006 (7)
S31	0.0343 (3)	0.0509 (3)	0.0531 (3)	0.0143 (2)	−0.0025 (2)	0.0033 (2)
C32	0.0275 (9)	0.0375 (10)	0.0317 (9)	0.0055 (8)	0.0045 (7)	−0.0035 (8)
N33	0.0261 (8)	0.0324 (8)	0.0330 (8)	0.0028 (6)	0.0036 (6)	0.0023 (6)
C34	0.0361 (11)	0.0364 (10)	0.0414 (10)	−0.0012 (8)	0.0067 (8)	0.0057 (8)
C35	0.0504 (13)	0.0384 (11)	0.0483 (12)	0.0074 (10)	0.0056 (10)	0.0097 (9)
N36	0.0262 (9)	0.0461 (10)	0.0622 (11)	−0.0022 (7)	0.0045 (8)	0.0047 (8)
S41	0.0633 (4)	0.0360 (3)	0.0516 (3)	−0.0144 (3)	0.0148 (3)	−0.0128 (2)
C42	0.0263 (9)	0.0302 (9)	0.0369 (10)	−0.0011 (7)	0.0029 (7)	−0.0014 (7)
N43	0.0302 (8)	0.0266 (7)	0.0352 (8)	−0.0018 (6)	0.0064 (6)	−0.0013 (6)
C44	0.0405 (11)	0.0370 (10)	0.0380 (10)	−0.0067 (8)	0.0096 (8)	0.0005 (8)
C45	0.0468 (13)	0.0361 (10)	0.0497 (12)	−0.0121 (9)	0.0086 (10)	0.0030 (9)
N46	0.0569 (12)	0.0439 (10)	0.0374 (9)	−0.0117 (8)	0.0147 (8)	−0.0049 (7)

### Geometric parameters (Å, °)

**Table:**

Cd—N13	2.3569 (14)	C25—H25	0.9300
Cd—N33	2.3886 (14)	N26—H26A	0.8600
Cd—N43	2.4308 (14)	N26—H26B	0.8600
Cd—N23	2.4432 (14)	S31—C35	1.711 (2)
Cd—Cl2	2.6294 (5)	S31—C32	1.7310 (19)
Cd—Cl1	2.6560 (4)	C32—N33	1.312 (2)
S11—C15	1.719 (2)	C32—N36	1.358 (2)
S11—C12	1.7420 (18)	N33—C34	1.384 (2)
C12—N13	1.312 (2)	C34—C35	1.335 (3)
C12—N16	1.340 (2)	C34—H34A	0.9300
N13—C14	1.387 (2)	C35—H35	0.9300
C14—C15	1.332 (3)	N36—H36A	0.8600
C14—H14A	0.9300	N36—H36B	0.8600
C15—H15	0.9300	S41—C45	1.721 (2)
N16—H16A	0.8600	S41—C42	1.7339 (18)
N16—H16B	0.8600	C42—N43	1.316 (2)
S21—C25	1.726 (2)	C42—N46	1.337 (2)
S21—C22	1.7341 (18)	N43—C44	1.387 (2)
C22—N23	1.316 (2)	C44—C45	1.332 (3)
C22—N26	1.344 (2)	C44—H44A	0.9300
N23—C24	1.388 (2)	C45—H45	0.9300
C24—C25	1.335 (3)	N46—H46A	0.8600
C24—H24A	0.9300	N46—H46B	0.8600
			
N13—Cd—N33	178.41 (5)	N23—C24—H24A	121.6
N13—Cd—N43	90.48 (5)	C24—C25—S21	109.85 (15)
N33—Cd—N43	90.07 (5)	C24—C25—H25	125.1
N13—Cd—N23	87.39 (5)	S21—C25—H25	125.1
N33—Cd—N23	92.07 (5)	C22—N26—H26A	120.0
N43—Cd—N23	177.85 (5)	C22—N26—H26B	120.0
N13—Cd—Cl2	91.13 (4)	H26A—N26—H26B	120.0
N33—Cd—Cl2	90.39 (4)	C35—S31—C32	89.29 (10)
N43—Cd—Cl2	87.53 (4)	N33—C32—N36	124.62 (17)
N23—Cd—Cl2	92.33 (4)	N33—C32—S31	114.17 (14)
N13—Cd—Cl1	90.66 (4)	N36—C32—S31	121.10 (14)
N33—Cd—Cl1	87.82 (4)	C32—N33—C34	110.08 (15)
N43—Cd—Cl1	93.36 (4)	C32—N33—Cd	129.62 (12)
N23—Cd—Cl1	86.84 (4)	C34—N33—Cd	119.74 (12)
Cl2—Cd—Cl1	177.991 (15)	C35—C34—N33	115.94 (19)
C15—S11—C12	89.30 (9)	C35—C34—H34A	122.0
N13—C12—N16	125.25 (17)	N33—C34—H34A	122.0
N13—C12—S11	113.89 (14)	C34—C35—S31	110.51 (16)
N16—C12—S11	120.83 (14)	C34—C35—H35	124.7
C12—N13—C14	110.17 (15)	S31—C35—H35	124.7
C12—N13—Cd	129.36 (12)	C32—N36—H36A	120.0
C14—N13—Cd	120.32 (12)	C32—N36—H36B	120.0
C15—C14—N13	116.44 (18)	H36A—N36—H36B	120.0
C15—C14—H14A	121.8	C45—S41—C42	89.42 (9)
N13—C14—H14A	121.8	N43—C42—N46	124.85 (17)
C14—C15—S11	110.20 (15)	N43—C42—S41	114.21 (14)
C14—C15—H15	124.9	N46—C42—S41	120.94 (14)
S11—C15—H15	124.9	C42—N43—C44	109.66 (15)
C12—N16—H16A	120.0	C42—N43—Cd	130.33 (12)
C12—N16—H16B	120.0	C44—N43—Cd	119.83 (11)
H16A—N16—H16B	120.0	C45—C44—N43	116.75 (18)
C25—S21—C22	89.25 (9)	C45—C44—H44A	121.6
N23—C22—N26	124.78 (16)	N43—C44—H44A	121.6
N23—C22—S21	114.54 (13)	C44—C45—S41	109.95 (15)
N26—C22—S21	120.69 (13)	C44—C45—H45	125.0
C22—N23—C24	109.57 (15)	S41—C45—H45	125.0
C22—N23—Cd	128.01 (11)	C42—N46—H46A	120.0
C24—N23—Cd	122.38 (11)	C42—N46—H46B	120.0
C25—C24—N23	116.79 (17)	H46A—N46—H46B	120.0
C25—C24—H24A	121.6		
			
C15—S11—C12—N13	−0.25 (15)	C35—S31—C32—N33	0.69 (15)
C15—S11—C12—N16	177.77 (17)	C35—S31—C32—N36	−175.75 (17)
N16—C12—N13—C14	−177.55 (19)	N36—C32—N33—C34	175.40 (18)
S11—C12—N13—C14	0.36 (19)	S31—C32—N33—C34	−0.9 (2)
N16—C12—N13—Cd	7.0 (3)	N36—C32—N33—Cd	−13.3 (3)
S11—C12—N13—Cd	−175.09 (8)	S31—C32—N33—Cd	170.40 (8)
N43—Cd—N13—C12	131.98 (16)	N43—Cd—N33—C32	47.14 (16)
N23—Cd—N13—C12	−48.20 (15)	N23—Cd—N33—C32	−132.74 (16)
Cl2—Cd—N13—C12	−140.48 (15)	Cl2—Cd—N33—C32	−40.39 (15)
Cl1—Cd—N13—C12	38.61 (15)	Cl1—Cd—N33—C32	140.51 (15)
N43—Cd—N13—C14	−43.08 (13)	N43—Cd—N33—C34	−142.28 (14)
N23—Cd—N13—C14	136.74 (13)	N23—Cd—N33—C34	37.84 (14)
Cl2—Cd—N13—C14	44.46 (13)	Cl2—Cd—N33—C34	130.19 (13)
Cl1—Cd—N13—C14	−136.45 (13)	Cl1—Cd—N33—C34	−48.91 (13)
C12—N13—C14—C15	−0.3 (2)	C32—N33—C34—C35	0.7 (2)
Cd—N13—C14—C15	175.60 (14)	Cd—N33—C34—C35	−171.58 (14)
N13—C14—C15—S11	0.1 (2)	N33—C34—C35—S31	−0.2 (2)
C12—S11—C15—C14	0.05 (17)	C32—S31—C35—C34	−0.27 (17)
C25—S21—C22—N23	−0.46 (15)	C45—S41—C42—N43	0.69 (15)
C25—S21—C22—N26	179.14 (17)	C45—S41—C42—N46	−179.30 (17)
N26—C22—N23—C24	−178.86 (18)	N46—C42—N43—C44	179.12 (18)
S21—C22—N23—C24	0.72 (19)	S41—C42—N43—C44	−0.87 (19)
N26—C22—N23—Cd	−1.1 (3)	N46—C42—N43—Cd	−5.8 (3)
S21—C22—N23—Cd	178.50 (8)	S41—C42—N43—Cd	174.18 (8)
N13—Cd—N23—C22	−64.98 (15)	N13—Cd—N43—C42	−105.72 (16)
N33—Cd—N23—C22	116.52 (15)	N33—Cd—N43—C42	72.79 (16)
Cl2—Cd—N23—C22	26.04 (15)	Cl2—Cd—N43—C42	163.17 (16)
Cl1—Cd—N23—C22	−155.79 (15)	Cl1—Cd—N43—C42	−15.03 (16)
N13—Cd—N23—C24	112.54 (14)	N13—Cd—N43—C44	68.91 (14)
N33—Cd—N23—C24	−65.97 (14)	N33—Cd—N43—C44	−112.58 (14)
Cl2—Cd—N23—C24	−156.44 (13)	Cl2—Cd—N43—C44	−22.20 (13)
Cl1—Cd—N23—C24	21.73 (13)	Cl1—Cd—N43—C44	159.60 (13)
C22—N23—C24—C25	−0.7 (2)	C42—N43—C44—C45	0.7 (2)
Cd—N23—C24—C25	−178.63 (15)	Cd—N43—C44—C45	−174.98 (15)
N23—C24—C25—S21	0.4 (2)	N43—C44—C45—S41	−0.2 (2)
C22—S21—C25—C24	0.04 (17)	C42—S41—C45—C44	−0.28 (17)

### Hydrogen-bond geometry (Å, °)

**Table:**

*D*—H···*A*	*D*—H	H···*A*	*D*···*A*	*D*—H···*A*
N16—H16A···N23	0.86	2.63	3.277 (2)	133
N16—H16A···Cl1	0.86	2.81	3.3903 (19)	126
N16—H16B···Cl2^i^	0.86	2.52	3.2941 (18)	151
N26—H26A···Cl2	0.86	2.41	3.1722 (17)	149
N26—H26B···Cl1^ii^	0.86	2.51	3.3300 (16)	161
N36—H36A···N43	0.86	2.61	3.324 (2)	142
N36—H36B···Cl1^iii^	0.86	2.63	3.3810 (18)	147
N46—H46A···Cl1	0.86	2.44	3.2135 (18)	150
N46—H46B···N36^iv^	0.86	2.56	3.417 (2)	177

Symmetry codes: (i) *x*+1, *y*, *z*; (ii) *x*, −*y*+1/2, *z*+1/2; (iii) *x*−1, *y*, *z*; (iv) −*x*, −*y*, −*z*.
